# Junctional Adhesion Molecule 2 Mediates the Interaction between Hatched Blastocyst and Luminal Epithelium: Induction by Progesterone and LIF

**DOI:** 10.1371/journal.pone.0034325

**Published:** 2012-04-12

**Authors:** Ren-Wei Su, Bo Jia, Hua Ni, Wei Lei, Shun-Li Yue, Xu-Hui Feng, Weng-Bo Deng, Ji-Long Liu, Zhen-Ao Zhao, Tong-Song Wang, Zeng-Ming Yang

**Affiliations:** 1 Department of Biology, Shantou University, Shantou, China; 2 College of Life Science, Northeast Agricultural University, Harbin, Heilongjiang, China; 3 College of Life Science, Xiamen University, Xiamen, Fujian, China; State Key Laboratory of Reproductive Biology, Institute of Zoology, Chinese Academy of Sciences, China

## Abstract

**Background:**

Junctional adhesion molecule 2 (Jam2) is a member of the JAM superfamily. JAMs are localized at intercellular contacts and participated in the assembly and maintenance of junctions, and control of cell permeability. Because Jam2 is highly expressed in the luminal epithelium on day 4 of pregnancy, this study was to determine whether Jam2 plays a role in uterine receptivity and blastocyst attachment in mouse uterus.

**Methodology/Principal Findings:**

Jam2 is highly expressed in the uterine luminal epithelium on days 3 and 4 of pregnancy. Progesterone induces Jam2 expression in ovariectomized mice, which is blocked by progesterone antagonist RU486. Jam2 expression on day 4 of pregnancy is also inhibited by RU486 treatment. Leukemia inhibitory factor (LIF) up-regulates Jam2 protein in isolated luminal epithelium from day 4 uterus, which is blocked by S3I-201, a cell-permeable inhibitor for Stat3 phosphorylation. Under adhesion assay, recombinant Jam2 protein increases the rate of blastocyst adhesion. Both soluble recombinant Jam2 and Jam3 can reverse this process.

**Conclusion:**

Jam2 is highly expressed in the luminal epithelium of receptive uterus and up-regulated by progesterone and LIF via tyrosine phosphorylation of Stat3. Jam2 may play a role in the interaction between hatched blastocyst and receptive uterus.

## Introduction

The effective reciprocal interaction between an implantation-competent blastocyst and the receptive uterus is a prerequisite for the success of implantation [1]. Embryo implantation starts with the physical interaction between the apical surface of the luminal epithelium and the trophoblast of the hatched blastocyst. Ovarian progesterone and estrogen play key roles in these processes. Preovulatory estrogen secretion induces epithelial cell proliferation on day 1 of pregnancy. Progesterone from the newly formed corpora lutea superimposed with ovarian estrogen secretion on day 4 directs stromal cell proliferation and epithelial cell differentiation, leading to uterine receptivity for implantation [2]. Based on the ability to support implantation of active blastocyst, the receptivity of an uterus can be designated as prereceptive, receptive and nonreceptive phases [3]. The receptive state of the uterus is defined as the limited time when the uterine milieu is favorable to blastocyst acceptance and implantation. In mice, the uterus becomes receptive on day 4 of pregnancy or pseudopregnancy and proceeds to the refractory state on day 5 [3]. In humans, implantation beyond the putative window of receptivity will lead to increased spontaneous abortions [4]. Up to date, the molecular basis underlying receptivity regulation remains poorly understood.

Junctional adhesion molecule (JAM) family consists of many members with similar structural characteristic and belongs to the immunoglobulin superfamily. There are three members of JAM family, including JAM1 (also known as JAM-A), JAM2 (also known as JAM-B) and JAM3 (also known as JAM-C) [5]. All of the JAM proteins have an extracellular domain containing two immunoglobulin-like domains, a single transmembrane segment and a short cytoplasmic tail with a PDZ-domain-binding motif (Phe-Leu-Val) [6]. JAM1 contains a single disulphide bridge in each immunoglobulin-like domain, whereas JAM2 and JAM3 contain two bridges in the C2-type domain, which may impart structural constraints [6].

Jam2 can perform its physiological functions through both homophilic and heterophilic interactions. A recombinant protein Jam2-Fc binding assay showed that Jam2 can form homodimers [7]. Beside itself, Jam is also a receptor of Jam through its first Ig-like fold [5]. Jam can interact with T, NK, and dendritic cells through Jam3 [8]. The Jam2/3 heterodimeris contributed to leukocyte extravasation to the skin and mediate cutaneous inflammation [9]. Soluble Jam2 could dissociate soluble Jam3 homodimers to form Jam2/3 heterodimers, suggesting the interaction between Jam2 and Jam3 is stronger than that between Jam3 and Jam3 [10]. Jam2 can also interact with other adhesion molecules, such as integrin α4β1 [11], which supports lymphocyte rolling and adhesion [12].

Based on our preliminary microarray data, Jam2 was highly expressed on days 3 and 4 of pregnancy in mouse uteri compared to day 5 of pregnancy (our unpublished data). Considering that the implantation window is open in this period, we assume that Jam2 may play a role during blastocyst implantation. We showed that Jam2 is highly expressed in luminal epithelium of day 4 pregnant uterus, and regulated by progesterone and LIF during blastocyst implantation.

## Materials and Methods

### Animals and treatments

Mature mice (Kunming White outbred strain) were caged in a controlled environment (14 h light, 10 h dark). All animal procedures were approved by the Institutional Animal Care and Use Committee of Xiamen University (XMUEA-0080).

To induce pregnancy or pseudopregnancy, adult female mice were mated with fertile or vasectomized males of the same strain by co-caging, respectively (day 1 = day of vaginal plug). On days 1 to 4, pregnancy was confirmed by recovering embryos from the oviducts or uteri. The implantation sites on day 5 were identified by i.v. injection of 0.1 ml of 1% Chicago blue dye (Sigma, St. Louis, MO) in saline.

For RU486 treatment, pregnant mice were injected s.c. with RU486 (25 mg/kg; Cayman Chemical, Ann Arbor, MI) in 0.2 ml sesame oil twice at 20:00 on day 2 and 08:00 on day 3, and uteri were collected for further analysis at 08:00 on day 4 of pregnancy.

Ovariectomized mice were treated with progesterone (1 mg/mouse) for 1, 3, 6, 12, 24 or 48 h 15 days after ovariectomy operation, respectively. To examine whether nuclear receptors of progesterone are involved in steroid hormonal regulation, ovariectomized mice were treated with RU486 (25 mg/kg) 1 h before progesterone injection. RU486 was dissolved in sesame oil and injected s.c.. Controls received the vehicle only. Uteri from these mice was collected and frozen into liquid nitrogen for further analysis.

To induce delayed implantation, pregnant mice were ovariectomized under ether anesthesia at 08:30–09:00 on day 4 pregnancy. Delayed implantation was maintained by daily s.c. injection of progesterone (1 mg/mouse; Sigma) on days 5–7. To terminate delayed implantation, progesterone-primed delayed-implantation mice were treated with estradiol-17β (25 ng/mouse, s.c.; Sigma) on day 7. The mice were sacrificed to collect uteri 24 h after estrogen treatment. Delayed implantation was confirmed by flushing blastocysts from one horn of the uterus.

Embryos at morula stage were collected at midnight on day 3 of pregnancy. Early and late blastocysts were collected at 08:00 and 22:00 on day 4 of pregnancy, respectively.

### Isolation of the luminal epithelial sheets

The luminal epithelial sheets were isolated as previously described [13], [14]. Luminal epithelium free of stromal and blood cells, and with very limited glandular epithelial contamination was isolated from the day 4 uteri by using mild enzymatic digestion and mechanical method. Briefly, mouse uteri on day 4 of pregnancy were isolated, cleaned, washed and cut into short fragments. After a digestion in the fresh medium (HBSS with antibiotics) containing 1.2 mg/ml dispase (Roche Diagnostics GmbH, Mannheim, Germany) and 10 mg/ml trypsin (Sigma) for 90 min at 4°C and 30 min at room temperature, luminal epithelium was isolated by mechanical method using a crooked needle. The purity of isolated epithelial sheets was examined with anti-pan-cytokeratine (a marker for epithelial cells) and anti-vimentin (a marker for stromal cells) by Western blot, respectively. In our isolated epithelial sheets, there were no detectable bands for anti-vimentin antibody although a strong band was seen with anti-pan-cytokeratine antibody (Figure S1), suggesting that isolated epithelial sheets were nearly free of stromal cells. Uterine tissues were washed with HBSS 3 times and cultured in Opti-medium (Invitrogen Corp., Carlsbad, CA) for 3 h, and then these tissues were treated with progesterone (1 µg/ml)or LIF (100 ng/ml, Sigma). Antagonist and inhibitor were added into culture medium 1 h before progesterone or LIF treatment. The final concentrations of RU486 and Stat3 inhibitor VI (S3I-201, Calbiochem) were 1 µM and 100 µM, respectively. After treatment, luminal epithelium sheets were collected for Western blot analysis.

### Electroporation

Luminal epithelial sheets isolated as above were suspended in 800 µl Opti-Medium in a 4 mm cup and then mixed with 20 µg of c-Stat3 vector or pcDNA3.1 vector. Electroporation was performed with BTX-830 generators according to the manufacturer's instruction (BTX Technologies, Hawthorne, NY) under the set of 540 V and 80 ms, and repeated 5 times at 1 Hz. Then epithelial sheets were cultured for 18 h and collected for further analysis.

### *In situ hybridization*

Total RNAs from the mouse day 4 uteri were reverse-transcribed and amplified with the corresponding primers (forward: 5′-CCCAAAGAAGACTACCTCCTCC-3′and reverse: 5′-TTCCAGACTTCGTGTTCATTG-3′). The amplified fragment was cloned into pGEM-T plasmid (pGEM-T Vector System, Promega, Madison, WI) and then amplified with the primers for T7 and SP6 to prepare the templates for labeling sense and antisense probes, respectively. Digoxigenin-labeled antisense or sense cRNA probe was transcribed in vitro using digoxigenin RNA labeling kit (Roche).

In situ hybridization was performed as described [15], [16]. Briefly, uteri were cut into 4–6 mm long pieces and flash frozen in liquid nitrogen, and then cut into 10 µm frozen sections, mounted on 3-aminopropyltriethoxy-silane (Sigma)-coated slides and fixed in 4% paraformaldehyde solution. All of the sections were counterstained with 1% methyl green and the positive signal was visualized as dark brown.

### Immunohistochemistry

Frozen sections were fixed in 4% paraformaldehyde solution for 1 h, washed in PBS 3 times, and treated with 1% Triton X-100 for 20 min. After washing, sections were blocked in 10% horse serum for 1 h at 37°C and incubated with goat anti-mouse Jam2 IgG (R&D system, Minneapolis, MN) at 4°C overnight. Then sections were incubated with biotin-coupled rabbit anti-goat IgG antibody (Vector Laboratories, Burlingame, CA) and alkaline phosphatase-coupled streptavidin (Vector Laboratories), respectively. The positive signal was visualized as red color by Vector Red according to the manufacturer's protocol (Vectastain ABC-AP kit, Vector Laboratories). Endogenous alkaline phosphatase activity was inhibited by supplementing 1 mM levamisole (Sigma) into Vector Red substrate solution.

For the fluorescent staining of mouse blastocysts, blastocysts flushed from day 4 pregnant uteri were fixed in 3.7% formaldehyde/PBS, washed in 0.1% BSA/PBS, and incubated with anti-JAM2 antibody (R &D Systems) overnight at 4°C. After washing in 0.5% Triton X-100/0.5% BSA in PBS, blastocysts were incubated with FITC-conjugated rabbit anti-goat antibody and counter-stained with DAPI for nuclei. The fluorescent signals were examined under a confocal microscopy.

### Real time RT-PCR

Total RNAs from mouse uteri were isolated using TRIzol reagent according to the manufacturer's instructions (Invitrogen Corp., Carlsbad, CA). mRNAs from 10 morula, early blastocyst or late blastocyst were extracted by Dynabeads mRNA DIRECT™ Kit according to the manufacturer's instructions (Invitrogen), respectively. cDNA was reverse transcribed using the ExScript RT Reagents Kit (Perfect Real Time; TaKaRa, Dalian, China). For real-time PCR, cDNA was amplified using SYBR Premix Ex Taq (TaKaRa) according to the manufacturer's instructions. After analysis using the ΔΔCt method, data were normalized to Rpl7 expression. Jam2 primer sequences used for real-time PCR were 5′-ATGCTGCTGCTGCTACACTACTT-3′ and 5′-TGACTTCTTGACGGTGGTCTTTT-3′; Rpl7 primers were 5′-GCAGATGTACCGCACTGAGATTC-3′ and 5′-ACCTTTGGGCTTACTCCATTGATA-3′.

### Western blot

Proteins were extracted from uterine tissues with lysis buffer [(50 mM Tris-HCl, pH 7.5, 150 mM NaCl, 1% Triton X-100, 0.25% sodium deoxycholate, 1 mM NaF, 2 mM Na3VO4, and complete protease inhibitor cocktail (Roche)]. The concentration of proteins was measured by BCA reagent (Applygen, Beijing, China). Proteins were run on a 10% PAGE gel and transferred onto nitrocellulose membranes. Nitrocellulose membranes were then blocked in 5% low-fat milk in PBST (0.1% Tween 20 in PBS) for 1 h, and incubated with goat anti-mouse Jam2 antibody (1∶1000; AF988, R&D system), rabbit anti-glyceraldehyde-3-phosphate dehydrogenase (GAPDH) antibody (1∶2000, sc-25778; Santa Cruz Biotechnology), rabbit anti-total Stat3 antibody (1∶1000; #9132, Cell Signaling Technology, Boston, MA) or rabbit anti-phosphorylation (Tyr 705) Stat3 antibody (1∶2000, #9131, Cell Signaling Technology) overnight at 4°C. After washing in PBST, the membranes were incubated in rabbit anti-goat antibody or goat anti-rabbit antibody conjugated with horseradish peroxidase (1∶5000, Thermo Fisher Scientific Inc., Waltham, MA) for 1 h, followed by three washes in PBST. The signals were detected by an enhanced chemiluminescence kit (Amersham Pharmacia Biotech, Arlington Heights, IL).

### Adhesion assay

Recombinant mouse Jam2 protein (rJam2, 50 µg/ml, 988VJ, R&D System) was coated onto the bottom of 96 well plates using ELISA-coating buffer according to the manufacturer's protocol (070018, CellChip, Beijing, China) at 4°C overnight. After rJam2-coated plates were blocked with 1% BSA at 37°C for 2 h, 30 blastocysts collected from uteri 8 h after delay implantation was activated by estrogen treatment were seeded into each well in 50 µl of KMSO medium. BSA was used for control. After 14 h culture, adhesion rate of blastocyst was examined by three independent persons. Five replicates were done and analyzed. For competitive assay, 2 µg of recombinant Jam2 or rJam3 (1213-J3, R&D System) were added into 50 µl KMSO medium before co-culture with blastocysts.

## Results

### JAM2 expression in mouse peri-implantation uterus

**Figure 1:**
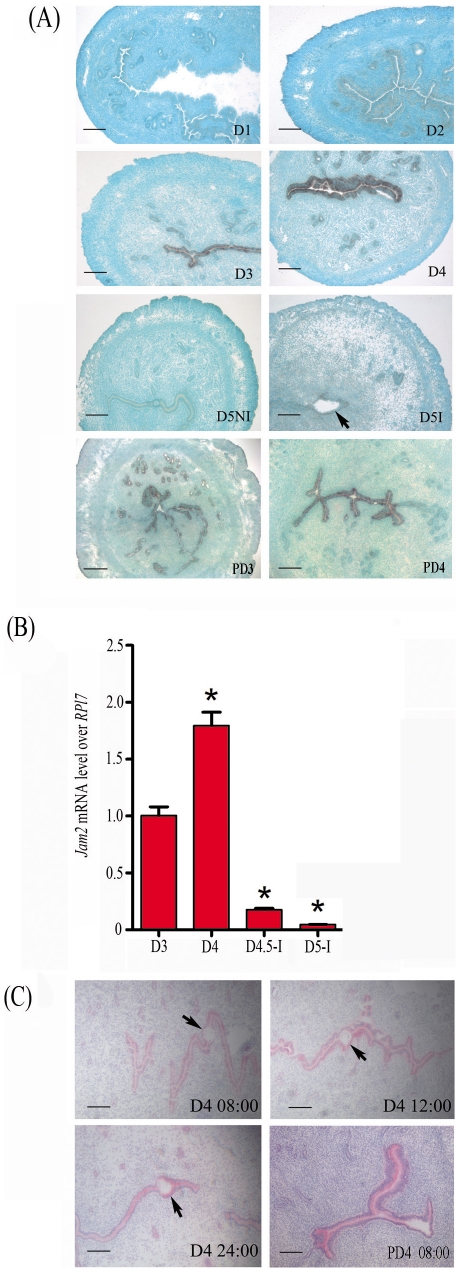
Jam2 expression in mouse uterus during early pregnancy. (A) In situ hybridization of Jam2 mRNA. (B) Real-time RT-PCR quantification of Jam2 mRNA. (C) JAM2 immunostaining. D1, day 1; D2, day 2; D3, day 3; D4, day 4; D4.5-I, implantation site at day 4 midnight; D4.5-NI, inter-implantation site at day 4 midnight; D5-I, implantation site on day 5; D5-NI, inter-implantation site on day 5; PD3, day 3 of pseudopregnancy; PD4, day 4 of pseudopregnancy; Arrow, embryo. Bar = 150 µm.

By in situ hybridization and immunohistochemistry, both Jam2 mRNA and protein were localized in the luminal epithelium. There was a high level of Jam2 mRNA signals in the luminal epithelium on days 3 and 4. The expression level of Jam2 mRNA was much lower from day 5 compared to day 4, but higher at implantation sites than at inter-implantation sites (Fig. 1A). Real time RT-PCR was used to confirm Jam2 mRNA level. Jam2 mRNA level was much higher on days 3 and 4 than that at day 4 midnight and on day 5 (Fig. 1B). Because Jam2 mRNA was highly expressed in the luminal epithelium on day 4 of pregnancy, immunohistochemistry was performed to examine whether JAM2 protein was expressed. From 08:00 to 24:00 on day 4 of pregnancy, JAM2 protein was detected in the luminal epithelium and weakly seen in the glandular epithelium (Fig. 1C).

We also checked Jam2 expression during pseudopregnancy to see whether Jam2 expression is dependent on the presence of embryos. Jam2 mRNA expression was strongly shown in the luminal epithelium on days 3 and 4 of pseudopregnancy (Fig. 1A). JAM2 immunostaining was also detected in the luminal epithelium on day 4 of pseudopregnancy (Fig. 1C).

### Progesterone regulation on Jam2 expression

**Figure 2:**
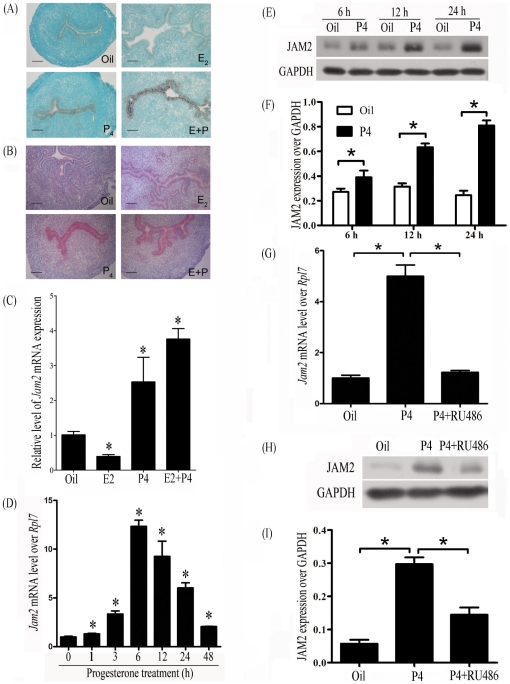
Progesterone regulation of Jam2 in the ovariectomized mouse uteri. (A) In situ hybridization of Jam2 mRNA expression after treatment with Oil (control), estrodiol-17β (E2), progesterone (P4) or a combination of estrogen and progesterone (E+P) for 24 h. (B) Immunostaining of JAM2 protein after treatment with steroid hormones for 24 h. (C) Real-time RT-PCR of Jam2 mRNA expression in uteri after ovariectomized mice were treated with Oil (control), estrodiol-17β (E2), progesterone (P4) or a combination of estrogen and progesterone (E+P) for 24 h. (D) Real-time RT-PCR of Jam2 mRNA expression after ovariectomized mice were treated with progesterone for 1, 3, 6, 12, 24 and 48 h, respectively. (E) A representative Western blot of Jam2 protein after ovariectomized mice were treated with progesterone for 6, 12 and 24 h, respectively. (F) The quantitative data in Fig. 2F. (G) Real time RT-PCR of Jam2 mRNA expression after ovariectomized mice were treated with progesterone alone or progesterone plus RU486 for 12 h. Sesame oil was served as control. (H) A representative Western blot of JAM2 protein after ovariectomized mice were with progesterone alone or progesterone plus RU486 for 12 h. P, progesterone; Oil, sesame oil. (I) The quantitative data in Fig. 2H.

Because Jam2 was highly expressed on days 3 and 4 of pregnancy and pseudopregnancy, ovariectomized mice were used to examine whether Jam2 expression is regulated by ovarian estrogen or progesterone. Adult ovariectomized mice were treated with estrogen, progesterone or estrogen plus progesterone for 24 h. A high level Jam2 mRNA and protein were detected in the luminal epithelium in progesterone group and estrogen plus progesterone group, but not in control and estrogen group (Fig. 2A, 2B). Data from real-time RT-PCR also confirmed Jam2 upregulation in progesterone group and estrogen plus progesterone group (Fig. 2C). To verify progesterone regulation, Jam2 expression levels following different time after progesterone treatment were quantified by real time RT-PCR. Jam2 mRNA level was significantly stimulated 1 h after treatment and reached the maximal level at 6 h (Fig. 2D). JAM2 protein expression was also up-regulated by progesterone treatment for 6, 12, and 24 h (Fig. 2E, 2F). RU486 was used to examine whether progesterone regulates JAM2 through progesterone receptor. Compared to control, the expression of both Jam2 mRNA and JAM2 protein was significantly up-regulated by progesterone treatment for 12 h, which was abrogated by RU486 treatment (Fig. 2G–I).

**Figure 3:**
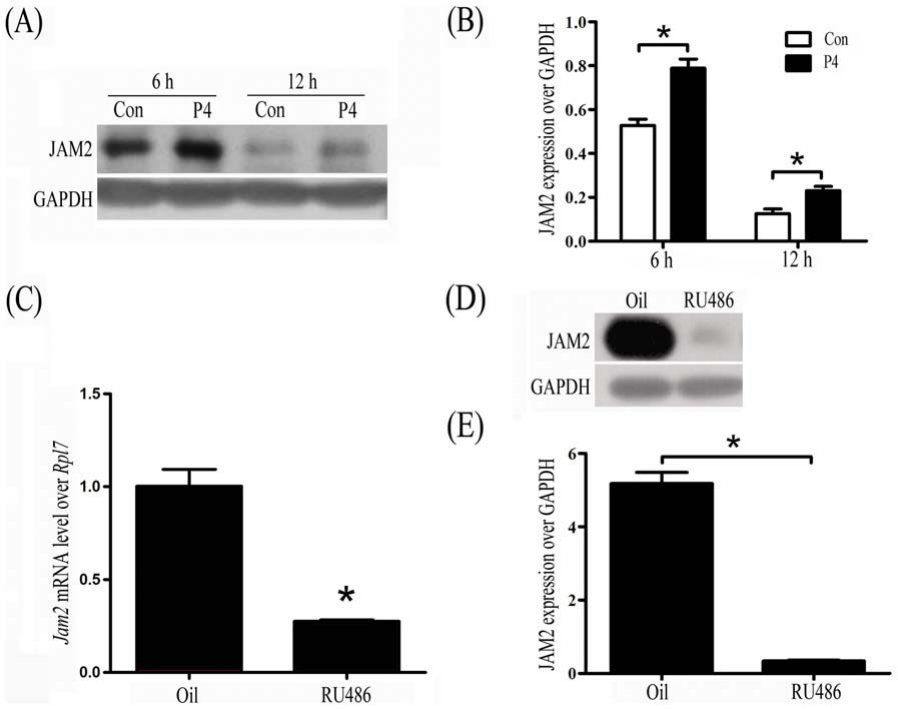
Progesterone regulation of Jam2 expression. (A) A representative Western blot of JAM2 protein after luminal epithelial sheets isolated from day 4 of pregnancy were treated in vitro with progesterone for 6 and 12 h, respectively. Progesterone was dissolved in ethanol. Ethanol was used as control. (B) The quantitative analysis in Fig. 3A. (C) Real time RT-PCR of Jam2 mRNA expression in mouse uterus on day 4 of pregnancy after pregnant mice were treated with RU486 (25 mg/kg) twice at 20:00 on day 2 and 08:00 on day 3. (D) A representative Western blot of JAM2 in mouse uterus on day 4 of pregnancy after pregnant mice were treated with RU486 (25 mg/kg) twice at 20:00 on day 2 and 08:00 on day 3. (E) The quantitative analysis in Fig. 3D.

Progesterone regulation on JAM2 was also determined in vitro. Luminal epithelial sheets isolated from day 4 uteri were cultured in Opti-Medium and treated with progesterone (1 µM). JAM2 expression in epithelial tissues was up-regulated by progesterone treatments for 6 and 12 h compared with control (Fig. 3A, 3B).

To check if JAM2 expression in vivo was also regulated by progesterone, pregnant mice were treated with RU486 (25 mg/kg) twice at 2000 on day 2 and 0800 on day 3. Both Jam2 mRNA and protein levels on day 4 were significantly down-regulated compared to normal pregnancy (Fig. 3C–E).

### JAM2 expression in luminal epithelium treated by LIF and Stat3 inhibitor S3I-201

**Figure 4:**
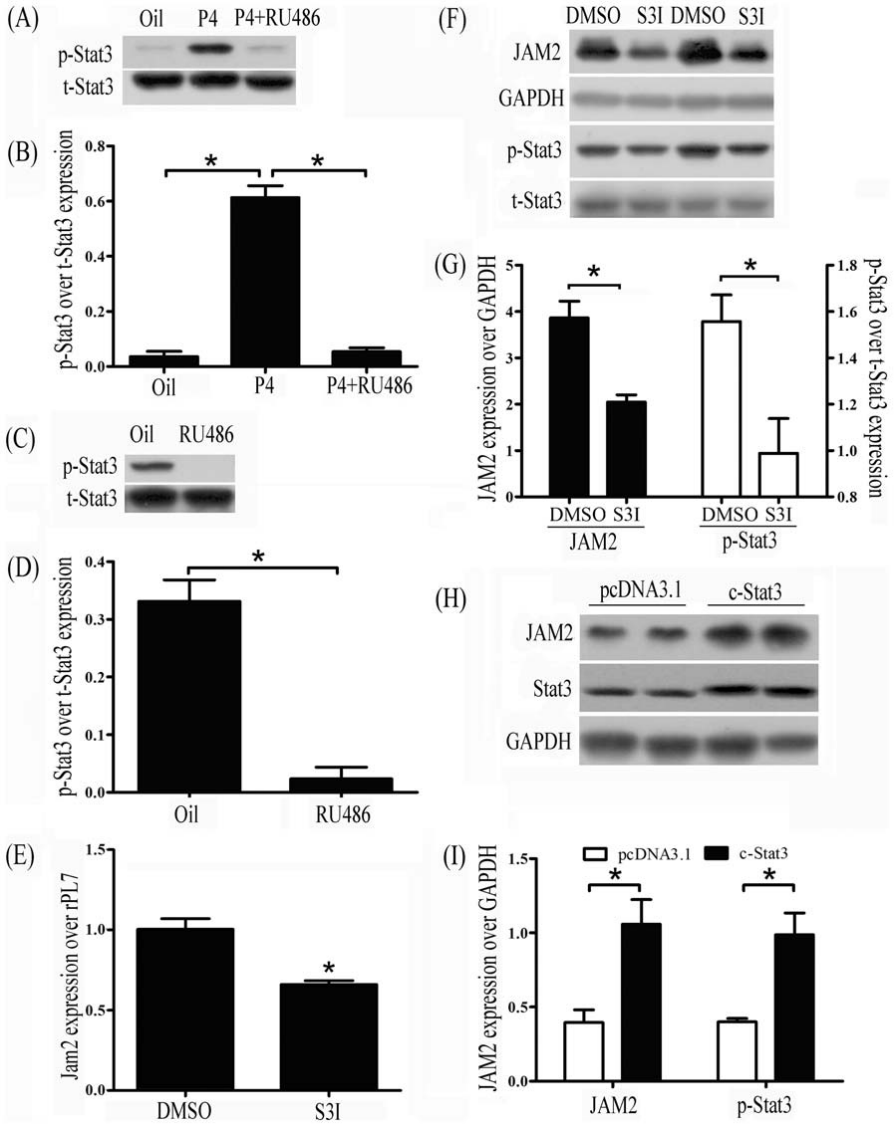
LIF and Stat3 regulation of Jam2 expression. (A) A representative Western blot of total and phosphorylated Stat3 in mouse uterus after ovariectomized mice were treated with progesterone (P) or a combination of progesterone and RU486. (B) The quantitative data in Fig. 4A. (C) A representative Western blot of total and phosphorylated Stat3 in mouse uterus on day 4 of pregnancy after pregnant mice were treated with RU486 (25 mg/kg) twice at 20:00 on day 2 and 08:00 on day 3. (D) The quantitative data in Fig. 4C. (E) Real time RT-PCR of Jam2 mRNA expression in mouse uterus on day 4 of pseudopregnancy after intrauterine injection of Stat3 phosphorylation inhibitor on day 3. (F) A representative Western blot of JAM2, total Stat3 and phosphorylated Stat3 in mouse uterus on day 4 of pseudopregnancy after intrauterine injection of Stat3 phosphorylation inhibitor on day 3. (G) The quantitative data in Fig. 4F. (H) A representative Western blot of JAM2 and Stat3 in cultured luminal epithelium after luminal epithelial sheets were transfected with c-Stat3 vector (c-Stat3, a continuous activated form of Stat3) and control vector (pcDNA3.1). (I) The quantitative data in Fig. 4H.

Since Stat3 phosphorylation at Tyr705 is at a high level in the luminal epithelium in the day 4 morning and essential for embryo implantation [14], [17], we assume that Stat3 may transcriptionally regulate Jam2 expression in the luminal epithelium. Therefore, we examined effects of progesterone on Stat3 phosphorylation. In ovariectomized mice, Stat3 phosphorylation was up-regulated 12 h after progesterone treatment, which was reversed by RU486 pre-treatment (Fig. 4A, 4B). In early pregnant mice, Stat3 phosphorylation was also inhibited by RU486 (Fig. 4C, 4D).

To examine Stat3 regulation on Jam2 expression, S3I-201 was injected into the uterine lumen on day 4 of pseudopregnancy. Three hours after injection, both Jam2 expression and Stat3 phosphorylation were significantly inhibited compared to control (Fig. 4E–G).

Vector c-Stat3 is a modified Stat3 expression vector expressing constitutively activated Stat3 [18]. When luminal epithelia isolated from day 4 of pregnancy were transfected with c-Stat3 vector through electroporation, the expression level of both JAM2 and Stat3 was up-regulated 18 h following electroporation compared to control vector pcDNA3.1 (Fig. 4H, 4I).

**Figure 5:**
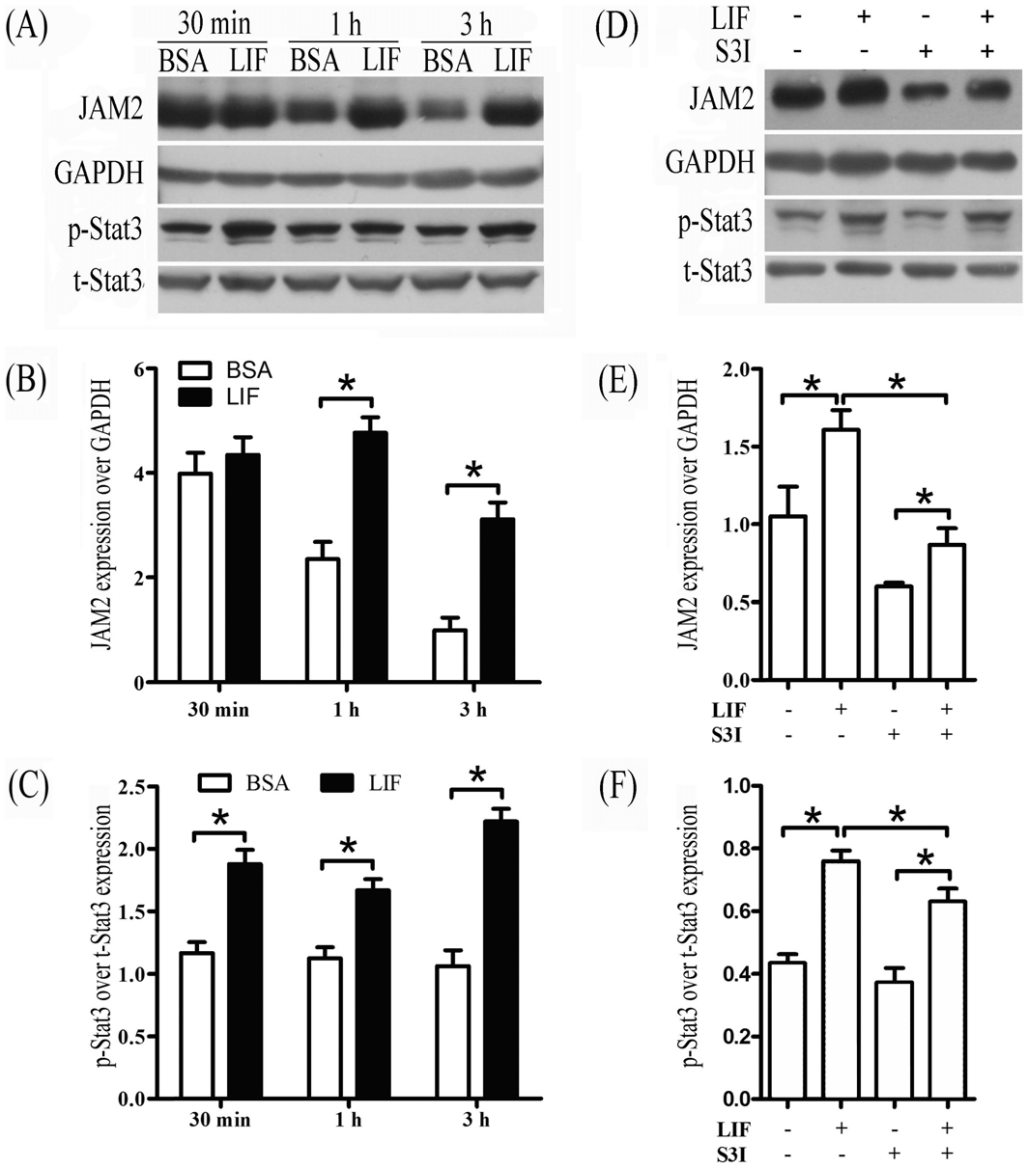
LIF regulation of Jam2 expression. (A) A representative Western blot of JAM2, total Stat3 and phosphorylated Stat3 in luminal epithelium after isolated luminal epithelial sheets were treated with LIF for 30 min, 1 h and 3 h, respectively. BSA was used as control. (B) The quantitative data of JAM2 in Fig. 5A. (C) The quantitative data of phospho-Stat3 in Fig. 5A. (D) A representative Western blot of JAM2, total Stat3 and phosphorylated Stat3 after isolated luminal epithelial sheets were treated with LIF with or without S3I for 3 h. (E) The quantitative data of JAM2 in Fig. 5D. (F) The quantitative data of phospho-Stat3 in Fig. 5D.

Because leukemia inhibitory factor (LIF) is highly expressed in mouse uterus on day 4 of pregnancy and essential for embryo implantation through phosphorylating Stat3 [14], [19]–[21], it is possible that LIF may also regulate Jam2 expression through Stat3 phosphorylation. Then we treated luminal epithelia isolated from day 4 uteri with LIF. Stat3 phosphorylation was up-regulated 30 min after LIF treatment, and JAM2 expression was up-regulated at 1 h and 3 h after LIF treatment (Fig. 5A–C). When luminal epithelia were treated with S3I-201, an inhibitor of Stat3 phosphorylation, LIF up-regulation on JAM2 expression was reversed (Fig. 4D–F).

### Jam2 expression in preimplantation embryos

**Figure 6:**
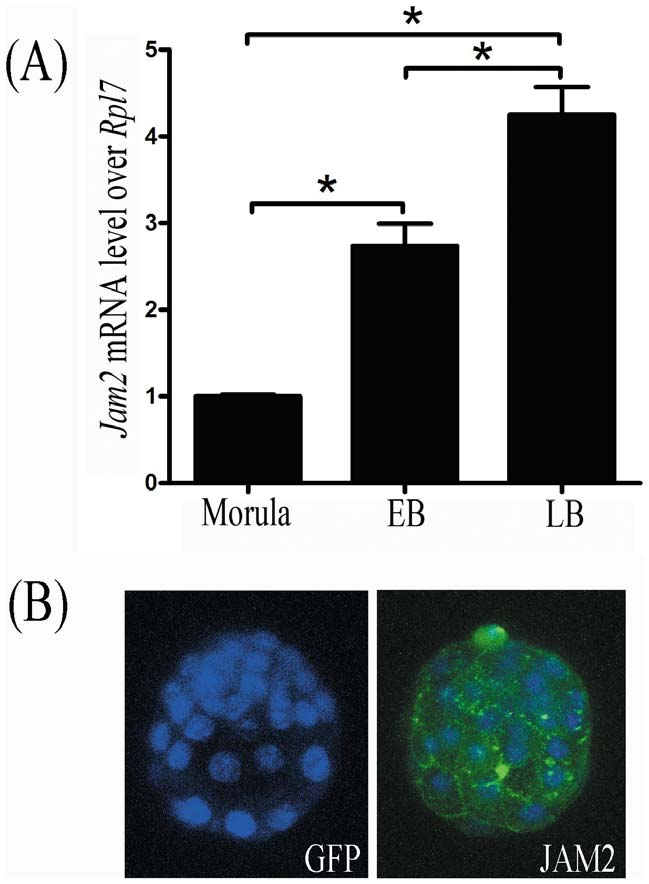
Jam2 expression in mouse embryos. (A) Real time RT-PCR of Jam2 mRNA expression in mouse embryos at morula, early (EB) and late blastocyst (LB) stages, respectively. (B) The immunofluorescent analysis of JAM2 protein in mouse blastocysts. Anti-GFP antibody was used as negative control. Blastocyst nuclei were counter-stained with DAPI (Blue staining).

Because Jam2 could mediate cell adhesion through forming homodimers with itself or heterodimers with Jam3 [5], real time RT-PCR was performed to check whether Jam2 and Jam3 are expressed in preimplantation embryos to mediate the adhesion between blastocysts and uterus. Jam2 mRNA expression was detected in morula, early blastocysts and late blastocysts, respectively. The trend of Jam2 expression in these embryos was as follows: late blastocyst>early blastocyst>morula (Fig. 6A). Although Jam3 was also detected in these embryos, there was no detectable difference among these embryos (data not shown). Immunofluorescence was also performed to examine JAM2 protein in mouse blastocysts. There was no detectable green fluorescent signal when anti-GFP antibody was used for a negative control (Fig. 6B). When anti-JAM2 antibody was used, the positive fluorescent signals were detected in the mouse blastocysts, especially at the cell junction zones (Fig. 6B).

### Blastocyst adhesion assay

**Figure 7:**
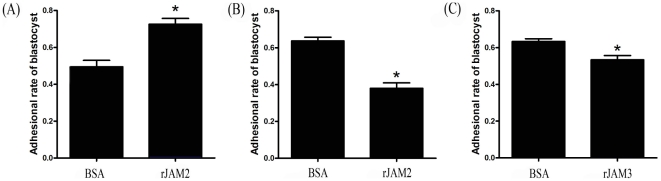
Blastocyst adhesion assay. (A) The adhesion rates of blastocysts on BSA or recombinant mouse Jam2 (rJam2)-coated dishes. (B) The adhesion rates of estrogen-activated blastocysts on the rJam2-coated dishes when cultured medium was supplemented with soluble rJam2 (40 µg/ml) or BSA. (C) The adhesion rates of estrogen-activated blastocysts on the rJam2-coated dishes when cultured medium was supplemented with soluble rJam3 (40 µg/ml) or BSA. BSA was used as a negative control.

Because Jam2 is an adhesion molecule and highly expressed in the luminal epithelium on day 4 of pregnancy, we suppose that JAM2 may play a role in the adhesion of blastocyst onto uterine luminal epithelium. Adhesion assay was performed to verify this hypothesis. Compared to BSA-coated plates, the rate of blastocyst adhesion on rJAM2-coated plates was significantly higher 14 h after blastocyst seeding (Fig. 7A). Because JAM2 can form either homodimer with JAM2 or heterodimer with JAM3, soluble recombinant JAM2 or JAM3 was used to challenge blastocyst adhesion in the JAM2-coated plates. Compared to BSA, blastocyst adhesion on JAM2-coated plates was significantly inhibited by either rJAM2 or rJAM3. However, the inhibition by rJAM2 was stronger than rJAM3 (Fig. 7B, 7C).

## Discussion

### Jam2 expression in the luminal epithelium may relate to uterine receptivity

Uterine receptivity is a restricted period when a uterus is receptive for blastocyst attachment [22]. In mice, the pre-receptive uterus on day 3 of pregnancy or pseudopregnancy becomes receptive on day 4 of pregnancy or pseudopregnancy and proceeds to the refractory state on day 5 [3]. Blastocyst transfer into intact pseudopregnant mice demonstrated that the window of implantation on day 4 remains open at least through 1800 h for normal day 4 blastocysts but only up to 1400 h for dormant blastocysts [23]. In this study, both Jam2 mRNA and protein were strongly expressed in the luminal epithelium on days 3 and 4 of pregnancy. Compared to day 3, Jam2 expression was much stronger in the luminal epithelium on day 4 of pregnancy. Additionally, Jam2 expression on days 3 and 4 of pseudopregnancy was similar to days 3 and 4 of pregnancy. These data suggest that Jam2 expression is closely related to uterine receptivity and is a potential molecular marker for uterine receptivity.

### Jam2 expression in mouse uterus is regulated by progesterone

Ovarian progesterone is essential for the success of implantation in mammalian reproduction [24]. Preovulatory estrogen can induce epithelial cell proliferation on day 1 of pregnancy and progesterone from newly formed corpora lutea initiate stromal cell proliferation from day 3. These coordinated effects of progesterone and estrogen direct stromal cell proliferation and epithelial cell differentiation for establishing the uterine receptivity [2].

In this study, Jam2 mRNA in ovariectomized mouse uterus was up-regulated by a single progesterone treatment or a combination of estrogen and progesterone. RU486, an antagonist of progesterone receptor, can abrogate Jam2 up-regulation by progesterone. Furthermore, Jam2 expression was down-regulated when pregnant mice were treated with RU486 on days 2 and 3. We also showed that progesterone could stimulate Jam2 expression in cultured luminal epithelial tissues. It has been reported that Jam2 was up-regulated in the luminal epithelium after ovariectomized mice were treated with a combination of estrogen and progesterone [25]. In humans, RU486 treatment can transform receptive endometrium into non-receptive phase [26]. These data suggested that progesterone might stimulate Jam2 expression in the luminal epithelium through nuclear progesterone receptor to prepare for receptive phase.

### LIF induces Jam2 expression in luminal epithelium via Stat3

LIF has been shown to be essential in initiating the blastocyst implantation process because LIF-deficient female mice are unable to accept implantation-competent blastocysts [21], [27]. A single injection of LIF into pregnant LIF-deficient females is sufficient to induce embryo implantation and normal development to term. Furthermore, injection of LIF could replace nidatory estrogen for inducing implantation [28]. During early pregnancy, LIF reaches the highest level on day 4 that just before embryo implantation [19], [20]. STAT3 can be specifically phosphorylated by LIF in the day 4 luminal epithelium [14]. Mice carrying a mutation of gp130, which delete all STAT-binding sites, are viable, but infertile in female mice because of implantation failure [29]. Before embryo implantation, functional blockade of Stat3 by injection into the uterine lumen of a cell-permeable Stat3 peptide inhibitor can specifically reduce embryo implantation by 70% [30]. Successful implantation is therefore dependent on phosphorylation and activation of Stat3 in the endometrium before implantation. In our study, Jam2 expression pattern in the luminal epithelium during early pregnancy is similar to that of LIF expression and phosphorylated Stat3 [14], [19], [20]. Therefore, we assumed that Jam2 expression should be regulated through LIF-Stat3 pathway.

Our in vitro culture model showed that LIF could induce Jam2 expression and Stat3 phosphorylation at Tyr 705 in cultured luminal epithelial tissues isolated from day 4 of pregnancy. Stat3 inhibitor VI(S3I-201) is a cell-permeable and specific inhibitor for Stat3 phosphorylation [18]. When isolated luminal epithelial tissues from day 4 uterus were treated with S3I-201, Jam2 expression was significantly reduced. Jam2 stimulation by LIF could be blocked by S3I-201 treatment. Furthermore, Jam2 expression was also increased by over-expressing a mutant Stat3 with constitutive activation. By injecting Stat3 inhibitor into uterine cavity on day 4 of pregnancy, we also found that the expression of Jam2 mRNA and protein in mouse uterus was reduced. Additionally, we also predicted Stat3 binding sites in the Jam2 promoter. Therefore, LIF up-regulates Jam2 expression in the luminal epithelium through Stat3 phosphorylation.

Furthermore, medroxyprogesterone acetate (MPA) can phosphorylate Stat3 at Tyr 705 and induce Stat3 nuclear localization in C4HD epithelial cells and T47D cell line of breast cancer, which can be reversed by RU486 [31]. In our study, progesterone is able to induce Stat3 phosphorylation in ovariectomized mouse uterus, which can be reversed by RU486. Stat3 phosphorylation in day 4 uterus was also significantly inhibited when pregnant mice were treated with RU486 on days 2 and 3. These data suggested that progesterone up-regulates Jam2 expression through the phosphorylation of Stat3. How progesterone regulates Stat3 phosphorylation remains to be further examined.

In the present study, we showed that Jam2 expression is up-regulated by progesterone and LIF. Jam2 expression on days 3 and 4 pregnancy is also correlated with rising levels of endogenous progesterone [2]. However, Jam2 expression on day 4 of pregnancy is stronger than that on day 3 of pregnancy. Because LIF expression on day 4 of pregnancy is mainly regulated by preimplantation estrogen surge [19], [20], [32], Jam2 expression on day 4 of pregnancy may be further stimulated by estrogen-regulated LIF. We did find that Jam2 expression in ovariectomized mouse uterus is strongly stimulated by a combination of progesterone and estrogen. It is possible that the high level of Jam2 expression in the luminal epithelium on day 4 of pregnancy is stimulated by the coordinated action of both progesterone and estrogen. However, Jam2 expression in ovariectomized mouse uterus was not induced by estrogen. Estrogen regulation of Jam2 in mouse uterus may require progesterone priming.

### JAM2 plays a role during blastocyst adhesion

In primates and rodents, embryonic implantation includes apposition, attachment, and invasion, leading to an effective interaction between the blastocyst and the maternal endometrium [3]. Roles of luminal epithelium are irreplaceable during these interactions, and many adhesion molecules in the epithelium are crucial during the establishment of uterine receptivity.

Our results showed that both Jam2 mRNA and protein were strongly localized in the luminal epithelium on day 4 of pregnancy, the day of uterine receptive phase, and in blastocysts, suggesting that Jam2 should play a role during the apposition and attachment phases. In our in vitro adhesion assay, recombinant mouse Jam2 protein could promote the adhesion between hatched blastocysts and Jam2-coated plates. This adhesion was partially inhibited by soluble rJam2 and rJam3, but the inhibition by rJam2 was stronger than rJam3. Although Jam2 could form homodimers with itself or heterodimers with Jam3 [5], the adhesion between blastocysts and receptive uterus should be mainly mediated by Jam2 because Jam3 was only weakly expressed in the luminal epithelium on day 4 of pregnancy. In this study, we showed that Jam2 is stimulated by LIF through Stat3. It is reported that LIF could increase the adhesion of endometrial epithelial cells via phosphorylated Stat3 [30]. It is possible that Jam2 may participate in the LIF-mediated the adhesion between maternal epithelial cells and blastocysts.

Another heterophilic binding interaction involved with Jam2 is the interaction between Jam2 and α4β1 integrin (VLA-4) [11]. VLA-4 is shown to be important in trophoblast binding to activated endothelial cells by antibody blocking [33]. In mice, α4 subunit expression is higher in blastocyst after hatching than before [34]. SGHPL-4, a human trophoblast-derived cell line, expresses α4β1 integrin and can adhere with endothelial cell line, SGHEC-7 [33]. These suggest another possibility that the attachment between blastocysts and receptive uterus may be also mediated by α4β1 integrin on blastocysts and Jam2 on luminal epithelium. However, Jam2 homozygous mutant mice are fertile and have no overt developmental defects [35]. Although *Jam2* gene is indeed disrupted in homozygous mutant mice, it is still possible that the transcript from the *Jam2* mutant locus may express certain JAM2 functions since the transcript contains a portion of the *Jam2* coding sequence [35]. It is also possible that Jam2 loss will lead to the compensation or up-regulation of other JAM family members.

In conclusion, Jam2 is highly expressed in the luminal epithelium and regulated by progesterone and LIF through Stat3 phosphorylation. Jam2 may play a role in mediating the adhesion between blastocysts and uterine luminal epithelium.

## Supporting Information

Figure S1 **Western blot analysis of Vimentin and pan-cytokeratin levels in the isolated endometrial cells.** Pan-cytokeratin protein is strongly detected in the luminal epithelial sheets, but not in the stromal cells. However, vimentin is strongly detected in the stromal cells, but not in the luminal epithelial sheets. β-actin was used for a loading control.
(EPS)
